# Subnational and Dynamic Conceptualisations of Planning Culture: The Culture of Regional Planning and Regional Planning Cultures in Finland

**DOI:** 10.1080/14649357.2021.1896772

**Published:** 2021-03-16

**Authors:** Eva Purkarthofer, Alois Humer, Hanna Mattila

**Affiliations:** a Department of Urbanism, TU Delft, Delft, Netherlands; b Department of Geography and Regional Research, Universität Wien, Wien, Austria; c Department of Built Environment, Aalto University, Espoo, Finland

**Keywords:** Planning system, planning practice, region, culturised planning model, change

## Abstract

This article furthers the unconsolidated theoretical discourse on planning cultures, focusing on the region as a highly dynamic planning scale. The article discusses regional planning cultures, distinguishing two meanings: *regional planning cultures in regions*, referring to regionally specific approaches visible in planning practice, and *cultures of regional planning*, referring to a shared, abstract understanding of regional planning. The article proposes a refined view on the “culturised planning model” (CPM) with the aim to advance from a static model towards a framework for understanding differences among planning cultures over time and between geographical contexts.

## Introduction

Comparative research on spatial planning has been criticised for emphasising the comparison of planning systems, defined by legal and administrative frameworks (Reimer & Blotevogel, 2012). Thinking in terms of planning systems entails a significant degree of abstraction and generalisation and is thus not sufficient to represent actual planning practices, which depend on many factors, including the leeway of actors in interpreting rules and regulations as they work within these systems as well as the influence of the broader societal context in which planning takes place. This criticism has become even more topical during the last decades, as it is increasingly acknowledged that, in addition to statutory planning instruments and administrative territories, spatial planning works through informal governance arrangements and in soft spaces (Allmendinger & Haughton, 2009; Purkarthofer & Granqvist, 2021), which typically are not reflected in the formal planning systems.

As a response to the dominance of system-centred approaches, the concept of planning cultures has emerged in the academic discourse (e.g. Knieling & Othengrafen, 2015; Othengrafen & Reimer, 2013; Sanyal, 2005). Although the idea of planning cultures has not matured into a comprehensive theory (Fürst, 2009, 2016), the concept is useful to convey the significance of choices, leeway and attitudes of actors in the planning process. At the same time, it acknowledges that the actions of individuals are framed by established practices and ways of doing things at organisational and societal levels. This article builds on the definition of planning culture “as the way in which a society possesses institutional or shared planning practices” (Knieling & Othengrafen, 2009a, p. 43). We understand planning culture as the “missing piece” in bridging approaches focusing on planning systems and planning practices. Both system-centred and practice-oriented research perspectives have provided valuable insights but have been criticised for their shortcomings. The concept of planning culture can be useful to acknowledge both systems and practices as elements shaping planning.

Yet we identify limitations in the current conceptualisations and the empirical studies building on planning culture. First, planning culture is predominantly understood as a country-specific concept, discussed at a national scale (Knieling & Othengrafen, 2009b; Sanyal, 2005; Stead et al., 2015). However, nation-specific conceptions do not provide a meaningful advancement to analyse planning practices, as they rely on describing the legal and administrative system, typically anchored to the nation state level. While some contributions acknowledge that planning cultures may differ at the regional level (Friedmann, 2005), empirical studies to date seldom go beyond discussing individual cases (Knieling & Othengrafen, 2009b) or they use local and regional examples to identify national differences in planning culture (Othengrafen, 2010; Peer & Sondermann, 2016).

Second, we regard current conceptualisations of planning culture, specifically the “culturised planning model” (CPM) (Knieling & Othengrafen, 2009b, 2015), as overly static. While changes regarding planning systems can typically be traced to specific legislative or administrative interventions, changes in planning cultures can occur gradually (Granqvist et al., 2021). Thus, conceptual models must be capable of capturing change in planning cultures. To address these limitations, we need a conceptualisation of planning culture that can describe, explain and visualise differences between geographical contexts and differences over time.

This article builds on empirical data from Finland and focuses on the regional level. The regional scale is under-researched, yet particularly interesting, as it remains heterogeneous, contested and debated in many countries (Harrison et al., 2021; Purkarthofer et al., 2021). Regional planning shows variety regarding governance arrangements and planning instruments (Smas & Schmitt, 2021). In Europe, this is partly fuelled by the European Union and the requirements of EU Cohesion Policy. In many countries, regional administration faced new tasks or fundamental reforms in the light of EU accession, which in some cases had implications for spatial planning at the regional level. Moreover, globally the importance of city-regions as scales for economic development and policy intervention is increasingly acknowledged (Rodriguez-Pose, 2008), leading to reforms of state administration as well as changes regarding regional planning.

Despite the role of planning culture in shaping planning practice, the cultural dimension is not frequently considered in reforms recently debated in many countries (Galland & Elinbaum, 2015). This is hardly surprising, given that scientific conceptualisations of regional planning culture remain simplistic, assuming variation between regions primarily in countries with a federal structure (Reimer & Blotevogel, 2012). However, a better understanding of regional planning cultures, especially regarding aspects of change, is crucial, following the argument that regions are “becoming” instead of just “being” (Paasi, 2009), and regional planning cultures are ever-changing.

This article builds on data obtained in 22 semi-structured expert interviews conducted in 2017 and 2018 in Finland. The interviewees represent a cross section of actors involved in planning at different spatial scales, including actors working at the national, regional and local level. Based on the empirical material, we ask how regional planning is understood and practiced in Finland, how regional planning culture can be conceptualised to reflect differences between geographical contexts as well as differences over time, and how the “culturised planning model” (CPM) can be developed further, to advance from a static model towards a framework for understanding change.

First, we summarise the theoretical debate on planning cultures. Subsequently, we introduce two conceptualisations related to regional planning culture and demonstrate their usefulness through empirical data from Finland. Next, we show how these conceptualisations acknowledge differences between geographical contexts over time and thus advance the CPM. Finally, we highlight future research agendas and limitations of our approach.

## Planning Culture: A Premature Concept in the Academic Debate

The idea that cultural factors are reflected in planning practice is not new and has been discussed, especially in the context of North American planning, from the 1960s onwards (Bolan, 1969; Friedmann & Weaver, 1979). Bolan (1969) voices the need to understand how urban government planning and decision-making occur and how the surrounding environment and social structures influence them. Grant (1994) argues that culture is a complex concept merging values, beliefs, economic and political structures. Some seminal writings focus on the role of individual actors and their social interactions in shaping local practices (Forester, 1993, 1999; Healey, 1993; Krumholz & Forester, 1990). Others describe local cultures at the organisational level or highlight the specificities of planning in certain cities or processes (Clavel, 2010; Hirt, 2005; Throgmorton, 1993).

In Europe, planning scholars began discussing planning cultures in the 1990s against the background of European integration and cross-border cooperation, which revealed differences regarding the tasks, perceptions, organisational structures, values and societal beliefs underlying planning (Keller et al., 1993). Sanyal (2005) explores differences in the behaviour of planners arising from planning cultures, understood as “collective ethos and dominant attitude of professional planners in different nations towards the appropriate roles of the state, market forces, and civil society” (p. 3). Despite two theoretical chapters (Castells, 2005; Friedmann, 2005), the contributions in the edited volume employ a varied and unsystematic understanding of planning culture, thus not significantly furthering the theoretical conceptualisation.

Knieling and Othengrafen (2009b) discuss planning cultures in Europe with the aim to “develop a theoretical basis and conceptual framework for a systematic analysis and comparison of different planning cultures” (p. xxviii). Although contributions build on different approaches to understand planning culture, the editors aim at a more systematic conceptualisation by introducing the “culturised planning model” (CPM). The CPM brings together Gullestrup’s (2009) concept of culture, distinguishing between a horizontal, vertical and temporal dimension, and Schein’s (2004) three levels of culture introduced in the context of organisational culture and leadership. Both models distinguish between visible layers on the surface and hidden layers underneath, claiming that an understanding of both is needed to grasp complex processes.

Taking these ideas into the context of urban and regional planning, Knieling and Othengrafen (2009b, 2015; Othengrafen, 2010) introduce the three-tiered triangle-shaped CPM: *Planning artefacts* refer to visible planning products, structures and processes and form the top layer. Examples of planning artefacts include the built urban structures, plans and policy documents, and general characteristics of the planning system including its laws, instruments and organisations. The *planning environment* constitutes the middle layer: assumptions, values and cognitive frames shared by planners. These include planning semiotics and semantics, objectives and principles, the scope and range of planning, and norms and rules. The *societal environment* forms the base layer of the CPM and subsumes the broader societal context with its norms, beliefs and perceptions affecting spatial planning. Factors shaping the societal environment include the orientation towards time, attitude towards nature, properties of the state and general characteristics of society. Knieling and Othengrafen understand planning artefacts as easily recognisable cultural elements, while planning environment and societal environment are more difficult to perceive. The CPM thus resembles the iceberg metaphor: a small part of the whole is visible, while vast parts are hidden beneath the surface.

During the last decade, the debate on planning cultures continued, partly fuelled by the publication of two special issues related to the subject (Levin-Keitel & Othengrafen, 2016; Nadin, 2012). While the concept of planning culture has been criticised for being ill defined, vague and not having matured into a theory that can be operationalised (Fürst, 2009; Taylor, 2013), the CPM is recognised as the most sophisticated model to conceptualise planning culture to date (Fürst, 2016), and appreciated as an alternative to comparative research focusing on planning systems (Nadin et al., 2018). Nonetheless, the model has been criticised for its shortcomings. The following paragraphs introduce five (partly interlinked) fundamental criticisms related to the concept of planning culture in general and the CPM in particular.

First, research on planning culture often addresses the national level^1^ and thus, while aiming to look beyond planning systems as a basis of characterization and comparison, does not successfully avoid the constraints of methodological nationalism (Reimer & Blotevogel, 2012; Sanyal, 2016). Assuming a uniform national (political) culture implies nationwide homogeneity regarding societal and planning culture, thus neglecting variation at the subnational levels (Reimer & Blotevogel, 2012). While the need to address regional and local planning cultures is sometimes acknowledged, case studies rarely look beyond nation states. Peer and Sondermann (2016) claim that local examples are occasionally used to identify national differences, drawing generalised conclusions from single empirical examples. Thus, there is a need to consider planning cultures at several scales while acknowledging the influence of the scale of analysis on the findings (Getimis, 2012; Valler & Phelps, 2018).

Second, the current conceptualisations of planning culture have not sufficiently addressed the dynamic interactions between systems-related and cultural elements, and thus have been unfit to explain change. While changes to the planning system may be relatively easy to implement and identify, it remains unclear how changes regarding planning culture are initiated and how they should be analysed (Reimer & Blotevogel, 2012).

This is not to say that change has never been addressed in relation to planning culture and the CPM. Othengrafen and Reimer (2013) claim that the CPM can identify endogenous changes within the observed culture and exogenous changes rooted in the surrounding environment. They acknowledge that internal change-initiating factors, potentially considered experimental at first, can ultimately influence perceptions and ideas about planning. They argue further that the customisation of existing structures, frames, and policies on the level of planning artefacts or planning environment does not necessarily affect the underlying core cultural traits of the societal environment. This can be observed, for instance, in the supposed convergence of planning in the context of Europeanisation (Knieling & Othengrafen, 2015; Stead, 2013). Although we do not disagree with these observations, we argue that the CPM in its current form can only be used to explain why change does *not* occur.

Third, the focus on planning culture has been criticised for obfuscating the role of individual behaviour in shaping planning practice. By addressing organisational or professional cultures, some interpretations of planning culture assume conformity of actors and thus overlook the ability of individuals to interpret rules and norms (Ernste, 2012). Ernste (2012) suggests that in order to understand the cultural aspects of spatial planning, a theoretical framework would need to account for both the institutional/structural side and the actor/agent side.

Fourth, the concept of planning culture is criticised as too complex and vague to be operationalised^2^ (Fürst, 2009, 2016; Reimer, 2016). The analytical value of the CPM is doubted due to the unclear attribution of elements to specific levels in the model. The CPM thus cannot be understood as an operational framework (Getimis, 2012) but rather as a “structured inventory of cultural influences on spatial planning” (De Olde, 2015, p. 8) aiming to sensitise research to cultural elements of the planning process (Othengrafen, 2012). These shortcomings are amplified by the unspecified causality between the levels in the model (De Olde, 2015).

Because of this conceptual fuzziness, the findings drawn from the model lead to rather general statements, highlighting for instance, bipolarities of unitary and federal states or more and less rule-obedient societies (Getimis, 2012). Moreover, due to the unclear operationalisation of the concept, the case studies and examples presented are diverse and often inconsistent in empirical analyses. Reimer and Blotevogel (2012), for instance, urge to move from “anecdotal storytelling” towards more systematic empirical research along defined analytical dimensions. Such empirical studies are necessary to assess the explanatory value of the planning culture concept and avoid arbitrary conclusions based on fuzzy concepts (Othengrafen & Reimer, 2013).

Fifth, we identify a lack of spatial awareness in the CPM regarding broader geographies and physical reality. While the built environment is treated as planning artefact in the model, landscape and spatial structure are not represented in the CPM. This is a fundamental shortcoming, considering that spatial circumstances might indicate why different planning approaches are taken. In other words, the specific way of doing things in a region might reflect the physical reality and its socio-spatial implications (e.g. regional accessibility, population density, urbanisation dynamics and inter-regional connectivity).

### Proposing a New Perspective on the “Culturised Planning Model”

**Figure 1.**
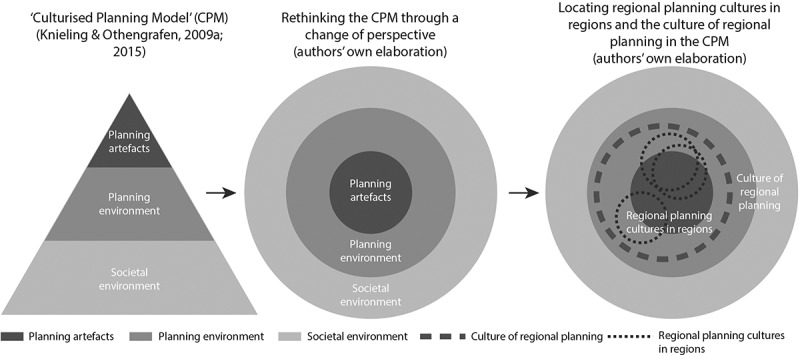
Rethinking the “culturised planning model” (CPM) to include *regional planning cultures in regions* and the *culture of regional planning.*

We recognise the potential of the CPM and its elements, yet we agree with many critical arguments raised. To develop the CPM further, we propose to distinguish two meanings of the term ‘planning culture’ at the regional scale: *cultures of regional planning* refer to a shared, abstract understanding of regional planning, while *regional planning cultures in regions* refer to regionally specific approaches visible in planning practice. The following sections elaborate on these meanings by presenting examples from regional planning practice in Finland. To integrate the two meanings into the CPM, we propose to switch the viewpoint: looking at the CPM “from above” turns the different layers into concentric circles (Figure 1).

We use the term *cultures of regional planning* to describe the conceptual understanding of the role of regional planning, capturing what regional planning can or should be, both in procedural terms (how it should take place) and substantive terms (what topics it should address). On the one hand, the cultures of regional planning are framed by the administrative context, which stipulates the responsibilities and competences of regional actors and the characteristics of regional planning instruments. On the other hand, the cultures of regional planning are affected by intangible factors such as values, customs and routines, as well as the perceived importance of regional planning. The cultures of regional planning reflect both planning-specific conventions and attitudes anchored in a broader societal context.

In terms of the CPM, we understand the cultures of regional planning to relate both to the planning environment and societal environment. The societal aspects include the general understanding of regions in state administration and roles of different government levels, as well as associations with “the regional”. The attitudes of regional actors are equally relevant for the culture of regional planning as are those of national or local actors.

We define the culture of regional planning as a dominating joint understanding, not necessarily shared by all, but largely accepted by societal actors. This understanding can be shared at the nation state level but might span other areas with shared societal or spatial characteristics, for instance, the Nordic countries, the American Midwest or sparsely populated regions. The culture of regional planning is not determined in individual regions but is a “common denominator” of planning practices across regions.

*Regional planning cultures in regions* refer to regionally specific approaches to planning, and showcase how planning is practiced. Different approaches and concrete actions are determined by the spatial reality of a region, the actors and organisations negotiating and implementing plans and policies, the formal and informal institutions they adhere to and the resources they have at their disposal.

Regional planning cultures in regions become visible at the intersection of planning artefacts and planning environment in the CPM. While not necessarily tangible in the physical world, they are manifestations of specific planning ideas in specific contexts. They are shaped by the planning environment but cannot simply be derived from it. In Finland, the Land Use and Building Act as core document of planning legislation states that “[i]t is the regional council’s function to carry out regional planning” (Land Use and Building Act, 1999, Section 19). However, while including general content requirements, the act does not specify which plan symbols are to be included in the regional land use plan or how often the plan should be renewed, let alone how strategic, restrictive or enabling the plan should be, thus leaving considerable room for different practices.

We suspect that the two meanings could be found at other spatial scales and in other planning contexts, for example, at the local or national level. We thus do not want to propose a “methodological regionalism” instead of a methodological nationalism, or claim that the regional scale is a superior level of analysis. Instead, we claim that while we might observe significant differences regarding planning practices within one planning system, at the same time a shared understanding of planning can continue to exist. This is relevant especially from the perspective of understanding differences in planning cultures over time, as well as specific cultural responses to different spatial realities.

## Cultures of Regional Planning in Finland

Based on our empirical data, we can identify a shared understanding of regional planning in Finland. Finland, like other Nordic countries, is a Unitarian state with strong local government (Sjöblom, 2010). With Finland’s accession to the EU in 1995, 18 regions were established, administered through Regional Councils. Their responsibilities include the formulation and enactment of regional land use plans and regional development programmes. The central state is represented at the regional level to advise and supervise planning through the Centres for Economic Development, Transport and the Environment (Puustinen et al., 2017). However, the understanding of regional planning is not directly derived from the planning system. While regional land use plans are plans of the hierarchically highest level, in practice municipal planning is considered more powerful in many respects (Hirvonen-Kantola & Mäntysalo, 2014). Regional planning often reacts to decisions taken at lower levels of planning rather than taking decisions itself (Mattila, 2018). Regional plans thus often reflect the sum of local plans and local interests, rather than steering local planning (Kilpeläinen et al., 2011). This is partly due to the weak political constitution of the Regional Councils, which are not independent authorities but joint municipal boards, consisting of local political representatives rather than directly elected officials (Purkarthofer & Mattila, 2018).

Another factor contributing to the imbalance lies in the frequent association of planning with the municipal scale and the understanding of land use plans as a local issue in the Finnish context (Purkarthofer, 2018), supported by the planning monopoly of municipalities, i.e. the right to be in charge of statutory land use planning within the municipal territory. Regional planning is more often associated with the provision of services and infrastructure to support economic regional development (Mattila, 2018).

In recent years, city-regional planning has gained importance, primarily in the form of contractual agreements regarding land use, housing and transport between the central state, the biggest cities and the surrounding municipalities (Bäcklund et al., 2018; Purkarthofer & Humer, 2019). The relationship between city-regional agreements and regional plans remains ambiguous, as one interviewee points out:

How can we do this thing together, these two levels that we are doing right here? Because we have the Land Use and Building Act, so that’s the background, that’s the base for what we are doing here in the Regional Council. But at the same time the same municipalities are preparing the MAL 2019 [city-regional plan]. (Interviewee 1, Uusimaa Regional Council)

Plans to reform the Finnish regions towards more independent administrative entities are currently under way but have met political resistance. Although providing social and health services has been the main driver for reform and the most controversial issue in the process (Humer & Granqvist, 2020; Kivelä & Moisio, 2017), the reform also contributed to putting the role of regional planning into question. While some interviewees regard regional planning as obsolete and almost irrelevant against the background of the municipal planning monopoly, others see potential in the direct election of regional representatives, for example, stronger commitment by politicians. One interviewee explains:

Some of these mayors, they say […] that there will be no regional planning in the future. But in the legislation we don’t see it, at least yet. […] I think we need regional land use planning somehow. I see many good things and many possibilities when we have more democracy in the future. (Interviewee 1, Uusimaa Regional Council)

Nonetheless, we see indicators for shared attitudes about regional planning among Finnish planning actors. The essential coordinating function of regional plans for nature conservation and transport planning is, for example, widely recognised, while simultaneously the importance and independence of local planning is acknowledged. The intention of having a shared way of ‘doing’ regional planning is manifested in a yearly event entitled “Regional Planning Days” [Maakuntakaavoituksen neuvottelupäivät] in which planning actors, concerned with the regional scale, come together to discuss relevant scientific and practical developments and ongoing projects. Although the event is organised by the Ministry of Environment, it represents a bottom-up knowledge exchange and brings together Finnish regional planners as a community facing common challenges.

Moreover, regions are often considered as a suitable scale to interact with the European Union and handle issues related to EU Cohesion Policy. Finland’s accession to the EU in 1995 highlighted inherent assumptions about regional planning and the shared perceptions of planning professionals, for example, the association of land use planning with the municipal or neighbourhood scale (Purkarthofer, 2018). This is partly rooted in the education of planners almost exclusively within the design-oriented architecture curriculum until recently (Eskelinen et al., 2000, p. 44).

With the establishment of regional administration, several disciplines such as environmental protection, regional development and regional land use planning became relevant at the regional level, thus suggesting a broader understanding of planning. However, cultural barriers between disciplines prevail (Eskelinen et al., 2000) and integration of policies at the regional level is not a given (Purkarthofer & Mattila, 2018). The strong association between regions and the EU becomes apparent in the title of the European Spatial Development Perspective (ESDP) (CEC, 1999) in its translation: “European regional planning and regional development guidelines” [Euroopan aluesuunnittelun ja aluekehityksen suuntaviivat].

## Regional Planning Cultures in Finnish Regions

The empirical data from Finland shows that different regional planning cultures in regions have emerged within the same framework laid out through planning law and administrative structure. One interviewee describes the ambiguity of the planning law as follows:

If you read the [Land Use and Building Act], you quite fast notice that it is pretty vague. […] It doesn’t fix anything. So everything is negotiable. And it’s funny to work in different regions in Finland because they understand the same words in really different ways. It becomes a cultural issue. […] Even within Finland. It is not the legislation, it’s a cultural issue. (Interviewee 2, Tampere city-region)

**Figure 2.**
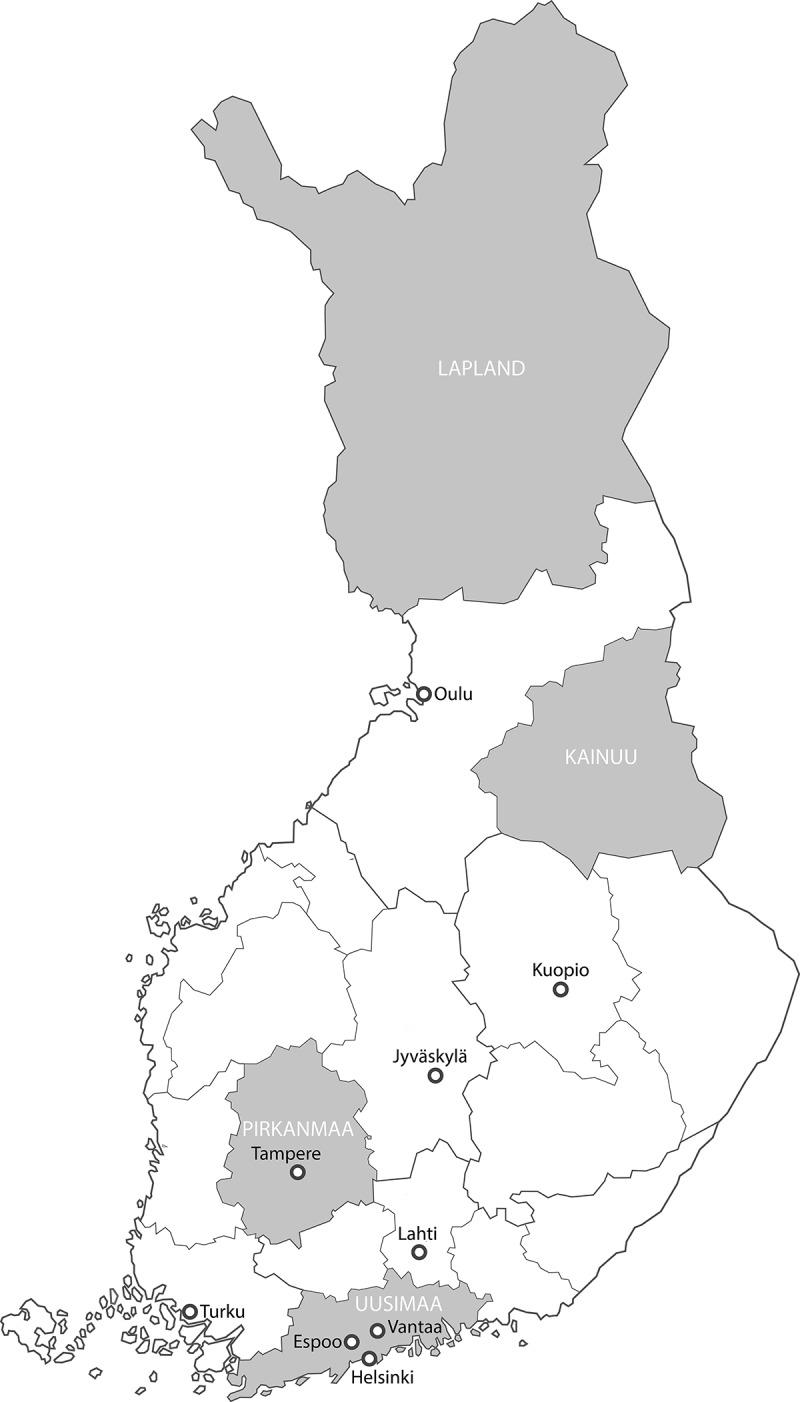
The administrative structure of Finland including cities over 100 000 inhabitants; the discussed regions are highlighted in grey

In this section, we present observations from four regions (Uusimaa, Pirkanmaa, Kainuu and Lapland, see Figure 2) to highlight fundamental differences regarding regional planning practices in Finland.

The region of *Uusimaa* consists of 26 municipalities, including three of Finland’s four most populous municipalities (Helsinki, Espoo and Vantaa). These cities are powerful actors in spatial planning, due to their financial and personnel resources and their political influence at the national level. The cooperation between municipalities in Uusimaa is sometimes challenging and has been further discouraged in the light of the regional reform and the introduction of mayors as political leaders in the country’s biggest cities, including Helsinki. Although Helsinki engages in strategic regional planning, the local objectives of the municipality overshadow regional cooperation (Granqvist et al., 2019). Moreover, some municipal actors are convinced that the regional level is not capable of addressing all aspects of planning:

The city has always been very strong in planning, so Helsinki does what it wants to do in a way. […] Helsinki is, because of its history and land ownership and so on, it is quite strong as an actor in planning. […] The region has no understanding of what creates value and what are the economic aspects of land use. (Interviewee 3, city of Helsinki)

In addition to the tensions between individual municipalities in the region, various sub-regional networks compete with each other. These include for instance, the state-led city-regional MAL planning work and agreement policy covering 14 municipalities (MAL); the “Helsinki Region Environmental Services” coordinating waste management and water services for four municipalities; the “Helsinki Region Transport” providing transport services and transport planning in nine municipalities; and the “KUUMA” network consisting of ten municipalities surrounding the metropolitan area. While some of these networks have formal competencies related to spatial planning, they also lobby for their interests in informal contexts and formal fora of decision-making. As these interests differ considerably, ranging from place branding and support for knowledge-intensive businesses at the core to the provision of attractive housing at the fringe, these activities potentially undermine efforts related to regional planning and development made by the Regional Council. Planning actors are aware of these tensions, as two interviewees highlight:

The relationship between the MAL [city-regional agreement], which is 14 municipalities, and the rest of the Uusimaa region is so difficult that Uusimaa has a major schizophrenia within the region itself. (Interviewee 4, city of Helsinki)

Uusimaa [Regional Council] does great work, a great job. But it has difficult relationships within, between its municipalities. (Interviewee 5, Pirkanmaa Regional Council)

As a result, regional planning in Uusimaa is often considered weak and not necessarily strategic. It has been argued, for example, that the regional council of Uusimaa has accommodated wishes from municipalities, resulting in “growth everywhere” policies (Mattila, 2018, p. 163). Because of these problems, Uusimaa Regional Council has been experimenting with new instruments:

The region of Uusimaa is currently drafting […] a general plan for the whole region, and then more detailed regional plans for the urban areas, metropolitan areas in particular. So, they have this idea: Okay, we can make this general regional plan as before, that’s fine, but we can also in the future make these metropolitan plans, directly influencing municipal plans. (Interviewee 6, Ministry of Environment)

In the region of *Pirkanmaa*, the regional land use plan is renewed every four years, similar to the “continuous master planning” which has gained popularity in some Finnish municipalities (Mäntysalo et al., 2019), and is used in the regional capital city Tampere. This four-year cycle aims to ensure that the planning process is turned into a continuous dialogue regarding strategic issues which is immediately linked to the election term of political decision makers, thus creating political interest and support for the plan.

The Pirkanmaa regional plan introduces “growth zones” [kasvu-vyöhykkeet] as unique map symbols intended to limit urban sprawl. The issue of dispersed settlements is especially important in Pirkanmaa, as the region consists of Tampere as central city and its surrounding municipalities. As smaller municipalities are prone to attracting “good taxpayers” with less restrictive land use policies, Tampere acknowledges the role of regional planning in these matters, as one interviewee highlights:

We have great relations [with the city of Tampere] and, for example, we are the only regional plan that got a statement from the main municipality that we should make the plan more binding than it currently is. I think this is very unique in Finland. (Interviewee 5, Pirkanmaa Regional Council)

The planning actors in Pirkanmaa believe they use the potential to make strategic choices in regional planning, and that they play a strong role in shaping the built environment, compared to other regions in Finland. They attribute the importance of the plan and the paucity of citizen appeals to a wide participation process involving municipalities, state actors and citizens.

However, tensions between city-regional and regional planning can be observed in Pirkanmaa. Actors from the Regional Council claim that the city-regional plans reveal the wishes of all municipalities but do not include strategic and potentially unpopular choices, which then need to be incorporated into formal regional planning:

It makes our work quite difficult of course. We are the bad guys. (Interviewee 5, Pirkanmaa Regional Council)

In the region of *Lapland* in Northern Finland, regional planning is understood and practiced differently. Lapland’s peripheral location, vast area and sparse population have led to declining population and jobs since 1990. These characteristics make it difficult to address challenges in municipal planning and have thus contributed to making regional planning in the region rather important:

In Lapland, regional planning is extremely important since we have vast areas of land. […] Tourism, forestry, mining, reindeer herding – you really need to accommodate different interests, and this works out very, very well in our regional planning processes. […] We are dealing with big issues, issues that cannot be dealt with in municipal plans. (Interviewee 7, Lapland Regional Council)

Despite the enormous land area covered by the region, the personnel resources in the public sector are limited. Actors working in the planning field in municipalities, the regional council or the state organisations in the region thus know each other on a personal level. These relationships affect their ways of working:

Lapland regional council is such a small organization. It is easy to discuss with each other. […] Lapland is a very compact region, even though it is a vast area. But people know each other. (Interviewee 8, Lapland Regional Council)

While actors in Lapland often feel alienated from the central government and the Helsinki capital region in the South of Finland, they consider themselves as part of the European Union and see an ally in the EU (Mattila et al., 2020). The support from the EU level includes both financial resources distributed to the region in the context of EU Cohesion Policy and conceptual and discursive influences related to urban and regional planning. One interviewee describes the influence of these ideas on the conception of regional planning, especially shortly after the establishment of Regional Councils in Finland:

The ESDP [European Spatial Development Perspective] and the concept of spatial planning were extremely important, especially when we started. They brought together many important fields of policy, like climate issues, transportation planning. All these things were nicely woven together. (Interviewee 9, Lapland Regional Council)

In the region of *Kainuu*, located in the North-East of Finland bordering Russia, regional planning is considered less important, although Kainuu, like Lapland, is sparsely populated. Since there is no population growth in the region, interviewees see little need for planning to steer development at the regional scale. According to one interviewee, most planning issues can be addressed at the municipal level:

There is more [on the webpage] about our regional planning, but we have the municipality level planning, which is much more sharp. (Interviewee 10, Kainuu Regional Council)

Between 2005 and 2012, Kainuu was the locus of a regional self-governance experiment, suggested by the Finnish government. The experiment temporarily altered the hierarchy of the state administration in the region, rescaling decision-making powers from municipal level (related to social and healthcare services) and state level (related to regional development) to the regional authority (Haveri et al., 2011). Moreover, direct regional elections provided democratic legitimacy to the Regional Council. While not specifically changing the status of regional planning, the experiment provided the opportunity to foster integration of different policies at the regional level and thus support a broader understanding of planning (Purkarthofer & Mattila, 2018). However, due to prevailing ambiguity regarding responsibilities and missed opportunities to make use of EU funding instruments, the conception of regional planning did not change as a result of the experiment. Instead, the experiment revealed and amplified tensions between municipalities in the region, which ultimately led to the discontinuation of the experiment in 2012.

As in the Lapland case, the actors in Kainuu are wary of their interests being sidelined in national politics and look to the EU for financial and political support:

The EU has been very useful, especially with a view to the tensions with the state level. […] If the EU support were lost, the Finnish state wouldn’t support the region. (Interviewee 11, municipality of Ristijärvi)

However, in Kainuu actors have been less successful in obtaining and utilising EU funding, and thus were not broadly able to operationalise EU instruments for regional planning and development.

These examples show that regional planning cultures in regions vary greatly, even within a centralised country such as Finland. Spatial realities become apparent as decisive factors in whether regional planning focuses on the management of rapid urbanisation, like in Uusimaa and Pirkanmaa, or on coping with sparsely populated and declining regions, like in Lapland and Kainuu. Relationships between various actors can render regional planning a mostly cooperative endeavour, observable for example, in Lapland, a situation dominated by competition, such as in Kainuu or Uusimaa, or a combination of both, as in Pirkanmaa.

Differences also become apparent in how the planning objective of growth is approached in the four regions. In Kainuu, with no growth to steer, regional planning is considered somewhat dispensable. In Lapland, regional planning pursues the goal of balanced development instead of strengthening the region’s biggest city. In Pirkanmaa, the goal at the regional scale is to enable growth but to limit it to desired areas. In Uusimaa, competing municipalities pursue their “growth everywhere” policies while regional planning does not have efficient instruments and political weight to manage rapid growth in the region.

## Advancing the “Culturised Planning Model” to Account for Differences over Time and Differences between Geographical Contexts

The distinction between *regional planning cultures in regions* and *cultures of regional planning* is not only useful to identify regional peculiarities and shared understandings, but especially to depict and explain changes of planning cultures. In this section, we present three examples of change derived from our insights into Finnish planning practice.

### Innovative Practices in Single Regions

**Figure 3.**
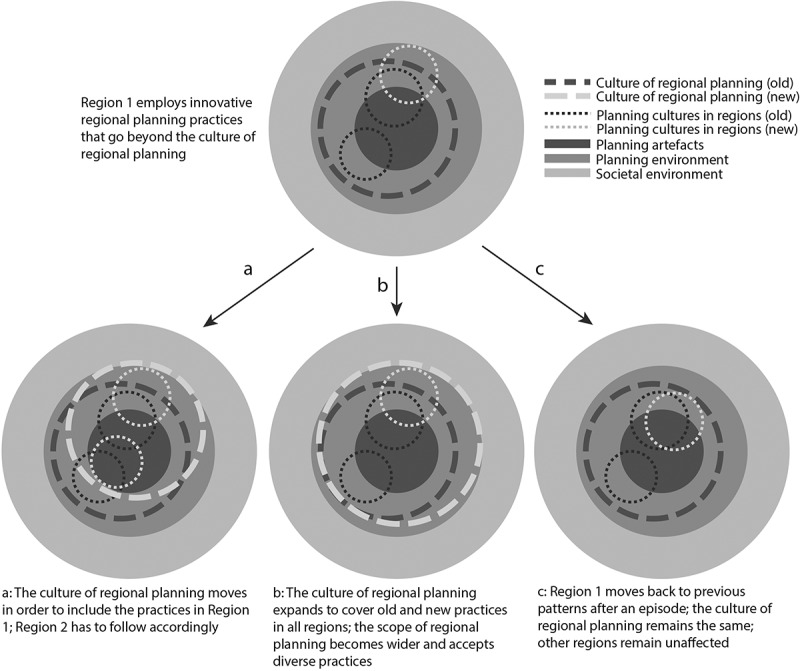
Innovative practices in one region can trigger various effects

One possible trajectory of change of planning culture is driven by the evolution of the regional planning culture in a specific region (Figure 3). Examples for such regional innovation could be the continuous four-year planning cycle in Pirkanmaa or the addition of city-regional plans developed by the Regional Council under discussion in Uusimaa. If one region incorporates new and innovative approaches into regional planning, its regional planning culture might go beyond the established *culture of regional planning*. In other words, planning practice in one region might incorporate new routines that were previously not considered to be within the regional planning “toolkit”. Over time, we envision three possible responses to such innovative practices.

First, these practices could become internalised into values that many planning actors hold, and come to affect the culture of regional planning shared by actors from other regions and society in general. Over time, other regions would be forced to adapt their practices accordingly, or at least justify why they do not apply commonly accepted practices (Figure 3, a). City-regional planning in Finland exemplifies such a transformation of the culture of regional planning. While initially practiced in a few regions, city-regional planning is being mainstreamed into planning practice, and potentially even anchored in the Finnish planning law if a current proposal for legal reform is enacted.

Many planning actors in Finland share the understanding that tackling challenges resulting from the growth of metropolitan areas is a key task for planning at the regional scale. This is a significant deviation from earlier attitudes about regional planning, which emphasised balanced development and safeguarding services and economic livelihoods throughout the country (Mattila et al., 2020). The task of regional planning in this context was often to address planning issues and regulate land uses in rural and peripheral areas for which no detailed master plan existed. While many now agree that city-regional coordination is crucial beyond the local scale, opinions differ as to whether Regional Councils should play a major role, or whether municipal planning departments should be responsible for city-regional planning in a collaborative manner.

Second, innovative practices in one region could dilute the culture of regional planning so that the understanding of regional planning broadens, and practices in regions diverge (Figure 3, b). The four-year cycle of developing and enacting the regional land use plan in Pirkanmaa is an example of such an expansion of the shared understanding. While some regions follow the cycle, others continue to renew their regional planning documents every ten or 15 years. Consequently, different attitudes linger in regions regarding the time horizon of regional planning. Nonetheless, we can identify shared understandings, too: No Finnish planning actor would consider one or two years as an appropriate time frame to renew plans, while probably no one would argue that plans should only be renewed every 25 years. Innovative practices regarding plan renewal in Pirkanmaa led to a broadening of the time horizon associated with regional planning, and any timeframe between four and approximately 15 years seems to be currently accepted by most planning actors.

Third, our “pioneering region” could abandon innovative practices after a relatively short time again. The planning culture in this region would then step back into the established culture of regional planning. The innovative practices thus represent a single episode but do not fundamentally change planning culture in the long run in this region, other regions or the shared culture of regional planning (Figure 3, c). The METKA project led by Uusimaa region between 2007 and 2008 is an example of such a short-lived change to a regional planning culture (Uusimaa Regional Council, 2008). The project aimed at increasing the ability to make strategic choices to enhance sustainable development with a view to the rapid urbanisation in the region. To do so, a collaborative working team consisting of representatives from several Regional Councils in Southern Finland, the regional state administration, road and rail administration and other relevant partners was established. The constructive and knowledge-intensive collaboration among this team of experts and public servants resulted in the development of the METKA-model aimed at densification of existing centres and the development of rail corridors between them (Dymén & Henriksson, 2009). However, the METKA project was not politically steered and the political decision-makers were not involved in the development of the model. Consequently, political priorities remained focused on regional policy instead of metropolitan policy, and the model did not directly shape regional or local plans in the end (Dymén & Henriksson, 2009).

While the METKA project brought about knowledge exchange between various actors, collaboration between the stakeholders was not continued in a similar format after 2008 and the challenges associated with urbanisation prevail in Uusimaa and other regions in Southern Finland. In terms of the CPM, no lasting change could be observed in Uusimaa, and instead, the region’s planning culture resumed its original state. As collaboration between multiple regions remains exceptional, the project did not alter the culture of regional planning in Finland.

### Changes in the Planning Environment

**Figure 4.**
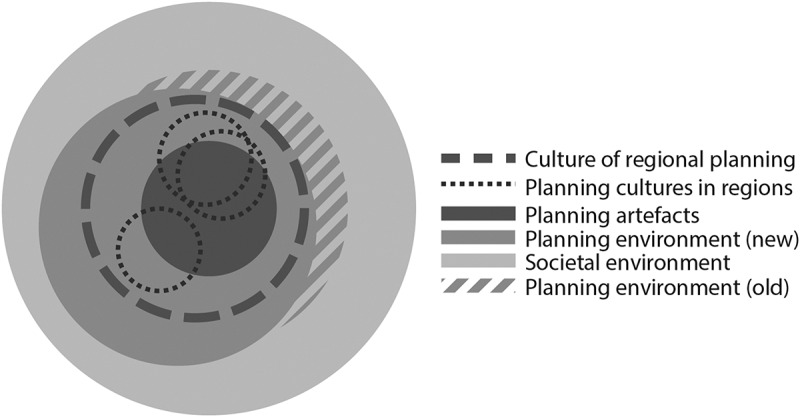
Changes regarding the planning environment do not necessarily change planning cultures

Changes to the planning environment, for example, regarding planning laws, do not necessarily lead to new practices if the culture of regional planning is not transformed accordingly (Figure 4). The self-government experiment in Kainuu region illustrates such a change in the planning environment. The experiment aimed to significantly strengthen regional self-governance through direct regional elections, added responsibilities for service provision and increasing self-determination regarding regional development. The changes also entailed possibilities to transform regional planning with a view to integrating regional development and regional land use planning. However, during the experiment the conception of regional planning remained narrow and the practices of regional planning did not change (Purkarthofer & Mattila, 2018). Consequently, viewed in the CPM, the experiment affected neither the culture of regional planning nor the regional planning culture in Kainuu directly. Significant legal and administrative changes at the level of the planning environment remained largely without consequences for planning practice.

### Changes to the Societal Environment

**Figure 5.**
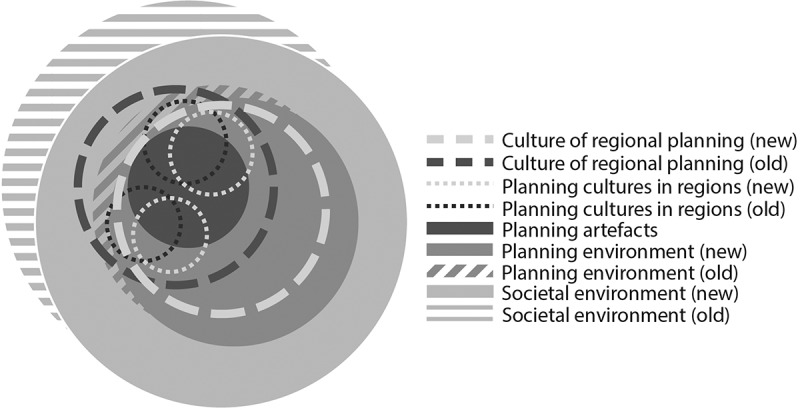
Structural changes to societal environment and planning environment affect the culture of regional planning; planning cultures in regions have to follow

Lastly, changes to regional planning cultures can be motivated by external or societal influences (Figure 5). The accession of Finland to the EU in 1995 and the resulting changes in administrative structures and tasks fundamentally affected the role and perception of regional planning (Fritsch & Eskelinen, 2011; Luukkonen, 2012). Through Regional Councils, the regions were formally established as part of the administrative system. Tasked with regional planning and regional development, the Regional Councils also assumed (partial) responsibility for the implementation of EU Regional and Cohesion Policy. In practice, this meant regional planning in Finland should conform with EU directives and guidelines (e.g. regarding environmental policy), apply for and implement EU funding instruments (e.g. regarding cross-border cooperation), and align objectives and strategies with EU policy documents (e.g. the Territorial Agenda) (Mattila et al., 2020).

EU Cohesion Policy also affected the values and expectations associated with regional policy. Following the ideals of the welfare state, Finnish regional policy traditionally emphasised balanced development throughout the country. Following EU priorities, regional policy is currently more concerned with juggling the objectives of competitiveness and cohesion, often favouring urban regions over peripheries (Mattila et al., 2020). Due to these structural changes at the societal and administrative level, regional planning cultures in regions as well as the culture of regional planning were forced to adapt and follow the direction provided.

Neither regional planning cultures in regions nor the culture of regional planning are static. Drivers for change can originate from either of the two. Our understanding of the CPM and conceptualisation of change resonate with what Healey (2004, 2006) discusses in the context of transformations of urban governance: pressure for change may come from single governance episodes, broader governance programmes or societal governance culture (Healey, 2006, p. 304). However, for a “real quantum transformation of governance […] all three levels need to change significantly” (Healey, 2004, p. 94). Similarly, contradictions between regional planning cultures in regions and the culture of regional planning cannot (permanently) exist. Thus, when a shift concerning either occurs, the resulting misfit creates pressure for change, as outlined above.

### Explanations for Change and Diversity: A Research Agenda

This article focuses on capturing the dialectic relationship between regionally specific practices and shared cultural understandings and on demonstrating how one can affect the other. However, space does not allow us to delve deeper into the explanations for change and diversity of planning cultures. We can identify several avenues for further research which could enrich the debate on planning cultures.

Planning cultures can be shaped by individual actors, their working practices and values. Research on structure and agency (Giddens, 1984; Jessop, 2001), discretion (Booth, 1996; Laws & Forester, 2015) and leadership (Sotarauta, 2016) can help to shed light on the behaviours of individuals, their job-related leeway and their professional and social skills. However, organisational cultures might be equally defining for regional planning cultures and thus require researchers to pay specific attention to the interactions among individuals, intangible rules, codes of conduct and established ways of doing planning. Taylor (2013) laid a promising foundation for future research by showing how historical and new institutionalist theories can enhance cultural analysis in planning. Planning research might also benefit from looking to scientific fields such as management studies and public administration to learn more about how organisations work and evolve.

Another major factor affecting planning cultures is learning. Planning education can play a decisive role in modifying the culture of regional planning by teaching future planners what (regional) planning is and how it is done. While planners might later question ideas communicated during their studies, such concepts might nonetheless form a base for their understanding of planning. The participation of planners in knowledge exchange activities (for example, international networks or working groups) contributes to policy mobility and learning. New ideas can be incorporated into local and regional planning cultures, while potentially being transformed to fit place-specific contexts (Healey, 2011).

Considering the importance of international organisations such as the EU in knowledge exchange activities, we could hypothesise that national contexts become less important in defining planning culture, and instead urban or peripheral regions tend to become more similar across Europe. However, such assumptions about convergence should be made with caution (Adams, 2008; Stead, 2013). Distinguishing between the *culture of regional planning* and *regional planning cultures in regions* can help to clarify which practices, behaviours and goals are shared and which are specific to one region. Hence it can be helpful to understand what regional planning in Uusimaa has learned from Stockholm, Berlin or Lombardy and the characteristics it shares with other Finnish regions.

## Conclusion

This article builds on the conceptual debate on planning cultures, which emphasises the need to understand intangible and sometimes invisible aspects shaping planning practices and systems. Planning culture has been criticised for being too vague to be operationalised (Abram, 2016; Fürst, 2009; Reimer, 2016) and current conceptualisations do not reflect differences over time and between geographical contexts. To turn planning culture into a sharper analytical concept, we distinguish between two different meanings related to the regional scale: *regional planning cultures in regions*, referring to regionally specific approaches of planning practice, and *cultures of regional planning*, summarising the shared understanding of what regional planning is and should do. We have linked these two conceptualisations to the “culturised planning model” (CPM) (Knieling & Othengrafen, 2009b, 2015), with the intention to advance the CPM from a static model to a framework capable of showing how misfits between the two concepts create pressure for change.

Empirical examples from Finland highlight noteworthy differences regarding planning practices at the regional level within one country and planning system, thus underscoring the significance of planning culture in understanding planning practice. These findings can contribute to overcome simplistic assumptions about (regional) planning culture, which associate variation with federal systems and uniformity with centralised systems.

Engagement with planning cultures can support practitioners’ “reflection-in-action” (Schön, 1983) by considering whether established ways of doing things are the result of individual skills and priorities, organisational practices or shared values and understandings among the profession or society. Moreover, planning culture can shed light on the beliefs guiding conduct within organisations and thus enable organisational learning (Argyris & Schön, 1978). The acknowledgement of regionally specific practices and their influence on the broader culture of regional planning highlights the transformative power of innovative and creative solutions. This can motivate individual planners to strive for improvements, empower them to embrace actor discretion in their daily work and support them in not being crestfallen by single unsuccessful attempts.

## Notes

1. Two noteworthy exceptions to national comparison are presented by Levin-Keitel and Sondermann (2014) and Valler and Phelps (2018).

2. Li et al. (2019) recently attempted to operationalise planning cultures through measuring and comparing planners’ attitudes towards risk, trust and co-operation in three European countries.
